# China's health national adaptation plan for climate change: action framework 2024–2030

**DOI:** 10.1016/j.lanwpc.2024.101227

**Published:** 2024-10-22

**Authors:** John S. Ji

**Affiliations:** Vanke School of Public Health, Tsinghua University, China

In September 2024, China made a significant step toward addressing the health impacts of climate change with the release of the *National Climate Change Health Adaptation Action Plan (2024–2030).* Led by the National Disease Control and Prevention Administration and endorsed by 13 government ministries, this plan reflects a coordinated national effort to mitigate climate-related health risks.^1^^,^^2^ Developing Health National Adaptation Plans (HNAPs) is an extension of National Adaptation Plans (NAPs). Its alignment with the *National Strategy for Climate Change Adaptation 2035* demonstrates China's commitment to integrating health into climate resilience frameworks, marking a pivotal moment in global climate adaptation following the health declaration at COP28 (Table 1).^3^

**Table 1:** China's national climate change health adaptation action plan (2024–2030).

I. Action strategy and objectives	**Strategy:** - Adhere to the principle of balancing mitigation and adaptation. - Integrate health adaptation concepts throughout the climate change policy system. - Uphold principles of proactive, scientific, systematic, and coordinated adaptation. - Focus on preventing health risks from climate change, strengthening health adaptation actions, and enhancing health promotion capabilities. - Establish early warning mechanisms as a precursor to adaptation linkage mechanisms. **Objectives by 2025:** - Improve multi-departmental collaboration mechanisms. - Build a framework for policies and standards related to climate change and health. - Strengthen monitoring systems for climate-sensitive diseases. - Develop evaluation indicators for climate change and health. - Complete the first round of assessments on health risks, vulnerabilities, and adaptation capacities. - Formulate a list of major scientific research needs for health adaptation. **Objectives by 2030:** - Establish a comprehensive policy and standard system. - Continuously enhance monitoring and early warning capabilities. - Mature the evaluation system for health risks, vulnerabilities, and adaptation capacities. - Significantly enhance adaptation capacities and climate resilience in key regions and sectors. - Build a supportive societal environment for health adaptation.
II. Key action tasks	The plan outlines **ten key tasks** to advance health adaptation in response to climate change: 1. **Improve multi-departmental collaboration mechanisms:** Establish an integrated system of planning, monitoring, early warning, assessment, and intervention. 2. **Enhance policies and standards:** Integrate policies across health, disease control, environment, water resources, and meteorology sectors. Develop comprehensive interventions and technical guidelines. 3. **Strengthen early warnings for climate-sensitive diseases:** Establish monitoring and early warning systems for diseases sensitive to climate change. Issue health advisories and preventive measures for extreme weather events. 4. **Enhance dynamic assessments:** Identify health risks and vulnerable populations. Develop localized risk assessment indicators and establish regular evaluation mechanisms. 5. **Improve risk prevention and intervention capabilities:** Integrate risk management into infrastructure projects. Enhance urban climate resilience and disaster prevention capabilities. 6. **Enhance health security capabilities:** Improve emergency response and medical rescue capabilities. Strengthen psychological intervention services for affected populations. 7. **Increase climate resilience of health systems:** Promote green, low-carbon healthcare institutions. Enhance resilience to extreme weather events. 8. **Create a supportive societal environment:** Strengthen public education and awareness. Pilot adaptation actions in cities, rural areas, communities, and key facilities. 9. **Accelerate technological innovation:** Advance research and development in adaptation technologies and health products. Promote smart applications of big data. 10. **Promote global action:** Deepen international cooperation. Participate in global public health governance and contribute to global adaptation efforts.
III. Safeguard measures	To ensure effective implementation, the plan proposes the following measures: 1. **Strengthen organizational leadership:** Incorporate health adaptation actions into important work content. Establish coordination mechanisms and clarify departmental responsibilities. Provincial disease control departments should formulate implementation plans based on local conditions. 2. **Provide support and assurance:** Increase support for actions related to health adaptation. Include monitoring, early warning, and laboratory construction in priority projects and information platform development. 3. **Strengthen team building:** Establish a national expert committee on climate change and health. Build technical alliances and enhance think tank capabilities. Conduct standardized skill training and practical drills. Provide social training to enhance education and outreach abilities of social groups and volunteers. 4. **Strengthen implementation guidance:** Disease control departments, in collaboration with relevant departments, should promote task implementation and ensure effective progress. Manage the entire process of health adaptation actions to comprehensively grasp task development and implementation effects.

China HNAPs takes a “balancing mitigation and adaptation” approach, focusing on reducing greenhouse gas emissions and preparing for the unavoidable impacts of climate change. Two key milestones—2025 and 2030—highlight the phased approach, with early goals centered on infrastructure and data collection, while later targets aim at refining systems for long-term adaptation. A key strength of the plan is its emphasis on safeguarding measures, such as strengthening governance and ensuring accountability, crucial for its execution and sustainability.

While the action plan acknowledges that climate change is a global challenge—manifesting in heatwaves, floods, cold spells, and typhoons—China's context adds further complexity. As one of the world's largest and most populous nations, China faces diverse climate risks across a vast geographic landscape, from arid deserts and high-altitude plateaus to coastal regions. The varying vulnerabilities of urban, peri-urban, and rural populations make it difficult to implement a one-size-fits-all approach. Climate-sensitive health risks include both infectious diseases, which thrive under changing environmental conditions, and non-communicable diseases (NCDs), exacerbated by the same climate stressors, creating a dual burden on health systems.^4^

The multitude of factors underscores the importance of tailored, locally relevant solutions to address China's specific vulnerabilities. While HNAPs provide a broad framework for climate adaptation, they often need to be adapted to local conditions. These plans can act as a springboard for local adaptations by offering structure, funding, and guidance. This helps local authorities develop targeted strategies while creating a foundation for implementation studies and evaluations. Such localized efforts ensure that national goals translate into meaningful, actionable solutions from the province to the community levels.

The leadership in climate-related health adaptation comes at a critical time. This action plan serves as a national milestone and contributes to global health resilience in the face of climate change.

## Declaration of interests

John S. Ji serves as an external academic advisor for the development of the action plan. Additionally, he has received funding for research and travel related to the World Health Organization Technical Advisory Group on Climate Health Ethics and has a technical service agreement with the WHO Western Pacific Regional Office (WPRO).
